# Cerebral artery stump syndrome: a comprehensive pooled analysis of 188 angiographically confirmed cases

**DOI:** 10.3389/fneur.2026.1840765

**Published:** 2026-07-07

**Authors:** Ping Lu, Ling-Yun Cui, Yi Jin, Zhao-Hui Tian, Wei Wang

**Affiliations:** 1 Department of Neurology, China-Japan Friendship Hospital, Beijing, China; 2 Department of Neurology, Beijing Chaoyang Hospital, Capital Medical University, Beijing, China

**Keywords:** angiography, carotid stump syndrome, cerebral artery stump syndrome, ischemic stroke, vertebral artery stump syndrome

## Abstract

**Introduction:**

Cerebral artery stump syndrome (CASS), a rare aetiology of ischemic stroke, consists of two subtypes: carotid stump syndrome (CSS) and vertebral artery stump syndrome (VASS). It causes recurrent ischemic events due to thrombus formation. However, selection of therapeutic strategies remains controversial, and large-sample systematic evaluations are lacking.

**Methods:**

Relevant literature from PubMed, Embase, and China Biomedical Literature Database (inception to 15 September 2025) were retrieved. Angiography-confirmed case reports and case series of CASS were included. Data on baseline characteristics, clinical features, treatment, and prognosis were extracted. Additionally, three typical cases from our institution were enrolled. Differences in clinical features and recurrence were analyzed. A two-tailed test was performed, with *p* < 0.05 considered statistically significant.

**Results:**

A total of 188 patients (CSS = 103, VASS = 85) from 43 studies were included. Therapeutic modality was the key factor associated with disease recurrence. The recurrence rate with medical treatment alone (19.0%) was significantly higher than that with invasive treatments, including carotid endarterectomy (1.7%), endovascular embolization (0.0%), external carotid stenting (0.0%), and endovascular recanalization (5.4%) (*p* = 0.015). In the medical treatment alone subgroup, the recurrence rate with initial anticoagulant therapy was 42.1%, which was substantially lower than that with antiplatelet therapy (93.0%).

**Conclusion:**

This study confirmed that embolus source occlusion is central to CASS treatment. Invasive treatment was associated with a lower recurrence rate than medical treatment alone, and anticoagulation was the first choice of medical treatment. Subtype-specific individualized diagnosis and treatment should be implemented for CSS and VASS. This study provides evidence for standardized clinical diagnosis and treatment of CASS.

## Introduction

Cerebral artery stump syndrome (CASS) is a rare but definite cause of cerebrovascular events. The residual vascular stumps of occluded arteries may serve as potential sources of emboli, leading to recurrent cerebrovascular events (1, 2). Carotid stump syndrome (CSS) and vertebral artery stump syndrome (VASS) are the main subtypes of CASS. Thrombi formed from the residual internal carotid artery stump at the proximal occluded segment and the residual vertebral artery stump at the distal occluded segment enter the intracranial cavity through anastomoses, such as the external carotid-internal carotid/ophthalmic artery and ascending cervical/deep cervical-vertebral artery, respectively, resulting in embolic stroke (3, 4).

Given the high recurrence and disability rates associated with CASS, timely diagnosis and standardized treatment following the onset of ischemic symptoms are crucial. After excluding other embolic causes, observation of turbulent blood flow at the arterial stump via cerebral arteriography is the gold standard for diagnosing CASS. Currently, a multidimensional system of therapeutic regimens for CASS has been developed, including medical interventions such as antiplatelet and anticoagulant therapies, as well as invasive treatments such as endovascular therapy and surgery. However, no consensus exists in the academic community regarding an optimal therapeutic strategy (5–9). Although numerous case reports and small-sample series have explored therapeutic strategies for CASS, the clinical selection of treatment regimens remains controversial, and no large-scale pooled analysis has evaluated the effectiveness of these therapeutic modalities.

Therefore, this study aimed to clarify the effectiveness of different treatment regimens by comprehensively analyzing the existing literature and systematically summarizing the treatment data of patients with CASS. Through this approach, we sought to provide an evidence-based foundation and directional guidance for clinicians to formulate individualized treatment strategies, standardize diagnosis and treatment processes, and carry out subsequent targeted research.

## Methods

### Data sources and search strategy

The search strategy covered relevant databases (PubMed, Embase, and China Biomedical Literature Database) and was based on the combination of the following medical subject headings and keywords: [“Carotid Stump Syndrome” OR “Vertebral Artery Stump Syndrome” OR “Stump Syndrome”]. The search period ranged from the inception of each database to 15 September 2025, with no language restrictions. In addition, we conducted a manual search to check the reference lists of all included studies and previous relevant reviews to supplement potentially missed literature.

### Inclusion and exclusion criteria

The inclusion criteria for study design were as follows: case reports, case series, narrative reviews, and systematic reviews containing case reports. The diagnosis of CASS in the study participants had to be confirmed via cerebral arteriography.

The exclusion criteria were: (a) literature without case report content, including reviews, letters to the editor, and commentaries without individual case reports; (b) missing key data, including follow-up time and recurrence outcome; and (c) overlapping patient data across different publications – in such cases, only the study with the largest sample size was included to avoid duplicate statistical analysis.

### Study selection and data extraction

The research team initially screened all retrieved literature by reviewing titles and abstracts and excluded those that did not meet the inclusion criteria. Articles that passed the initial screening were further reviewed in full text, and the final list of studies included in this study was determined by strictly applying the inclusion and exclusion criteria. Literature screening was independently performed by two researchers (LP and WW). In case of disagreements during the screening process, a consensus was reached through joint consultation. A standardized table was used to extract the present data. The extracted study and patient characteristics were as follows: (a) literature information: publication year and number of case reports; (b) baseline characteristics: sex and age; (c) disease-related characteristics: stump type, location of vascular occlusion; (d) clinical symptoms: frequency of symptom onset, amaurosis fugax, vertigo, hemianopsia, ataxia, and consciousness disturbances; (e) comorbidities: hypertension and diabetes mellitus; (f) treatment regimens: antiplatelet drugs, anticoagulant drugs, endovascular recanalization, endovascular embolization, endovascular stenting, and carotid endarterectomy; and (g) outcome indicators: follow-up duration and recurrent cerebrovascular events. All enrolled patients were diagnosed with chronic arterial occlusion rather than acute tandem occlusion. This determination was based on typical manifestations including characteristic eddy currents and stagnant blood flow at the distal segment of the occluded vessels, well-established collateral circulation, as well as the clinical course of delayed and recurrent ischemic events, all of which are typical features of long-standing vascular lesions.

### Outcome definition

Recurrence was defined as the reappearance or deterioration of previous neurological symptoms and the identification of new lesions in the same arterial territory on follow-up neuroimaging (CT or MRI).

### Typical cases

This pooled analysis included three typical CASS cases (two CSS, one VASS) from the China-Japan Friendship Hospital. All diagnoses and treatment processes were in accordance with clinical standards and complete data records. The cases met the inclusion criteria after evaluation by two independent reviewers, and informed consent was obtained from patients or their family members.

### Statistical analysis

Continuous variables are expressed as medians and interquartile ranges, and categorical variables are presented as frequencies (percentages). Analysis of variance or the Wilcoxon rank-sum test was used to compare differences in continuous variables among groups, and Pearson’s chi-square test was used for categorical variables. A two-tailed test with *p* < 0.05 was considered statistically significant. All statistical analyses were performed using the IBM SPSS Statistics version 23 software (IBM Corp., Armonk, NY, United States).

## Results

### Typical cases

#### Case 1

**Figure 1:**
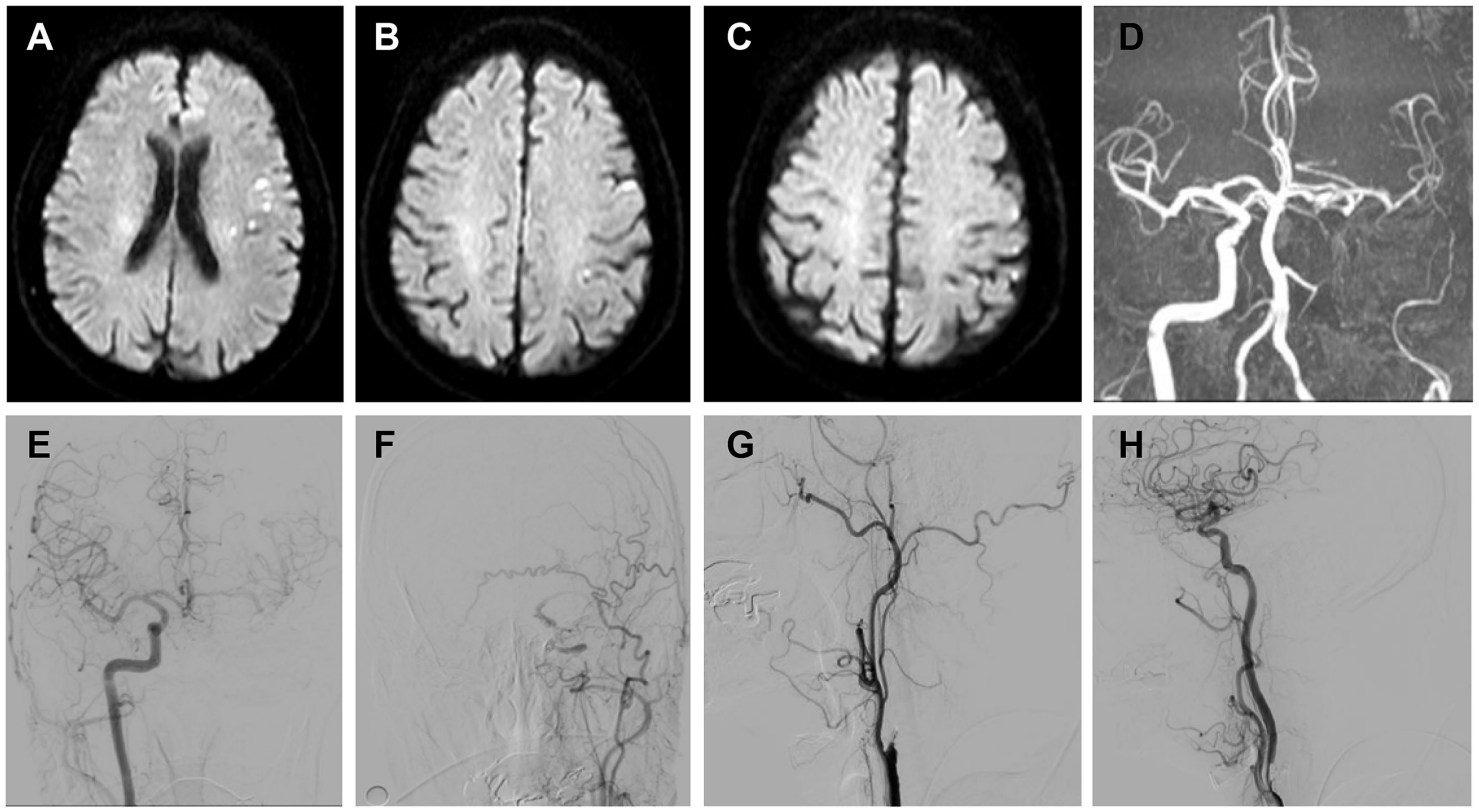
Imaging findings of Case 1. **(A–C)** Diffusion-weighted imaging showing scattered infarcts in the left frontoparietal lobe and insula. **(D)** MRA showing no visualization of the left ICA. **(E–H)** Digital subtraction angiography showing unobstructed blood flow at the ICA stump with local turbulence; the distal vessel remains completely occluded.

A 74-year-old male patient with hypertension, diabetes mellitus, and coronary heart disease presented with dizziness and numbness in the right limbs. Magnetic resonance imaging (MRI) revealed multiple cerebral infarctions in the left frontoparietal lobe and insula. Magnetic resonance angiography (MRA) confirmed complete occlusion of the origin of the left internal carotid artery (ICA) with a small residual stump. Dual antiplatelet therapy (aspirin 100 mg and clopidogrel 75 mg once daily) was started. Catheter angiography showed unobstructed blood flow at the left ICA stump with local turbulence; the distal vessel was completely occluded. The presence of well-established collateral flow via the ophthalmic artery and the absence of acute intraluminal thrombus beyond the stump were consistent with chronic ICA occlusion. Cardiac examinations ruled out cardiogenic embolism. A diagnosis of CSS was made. The family chose conservative treatment. At discharge, symptoms had improved. At 3-month follow-up, no recurrent stroke occurred (Figure 1).

#### Case 2

**Figure 2:**
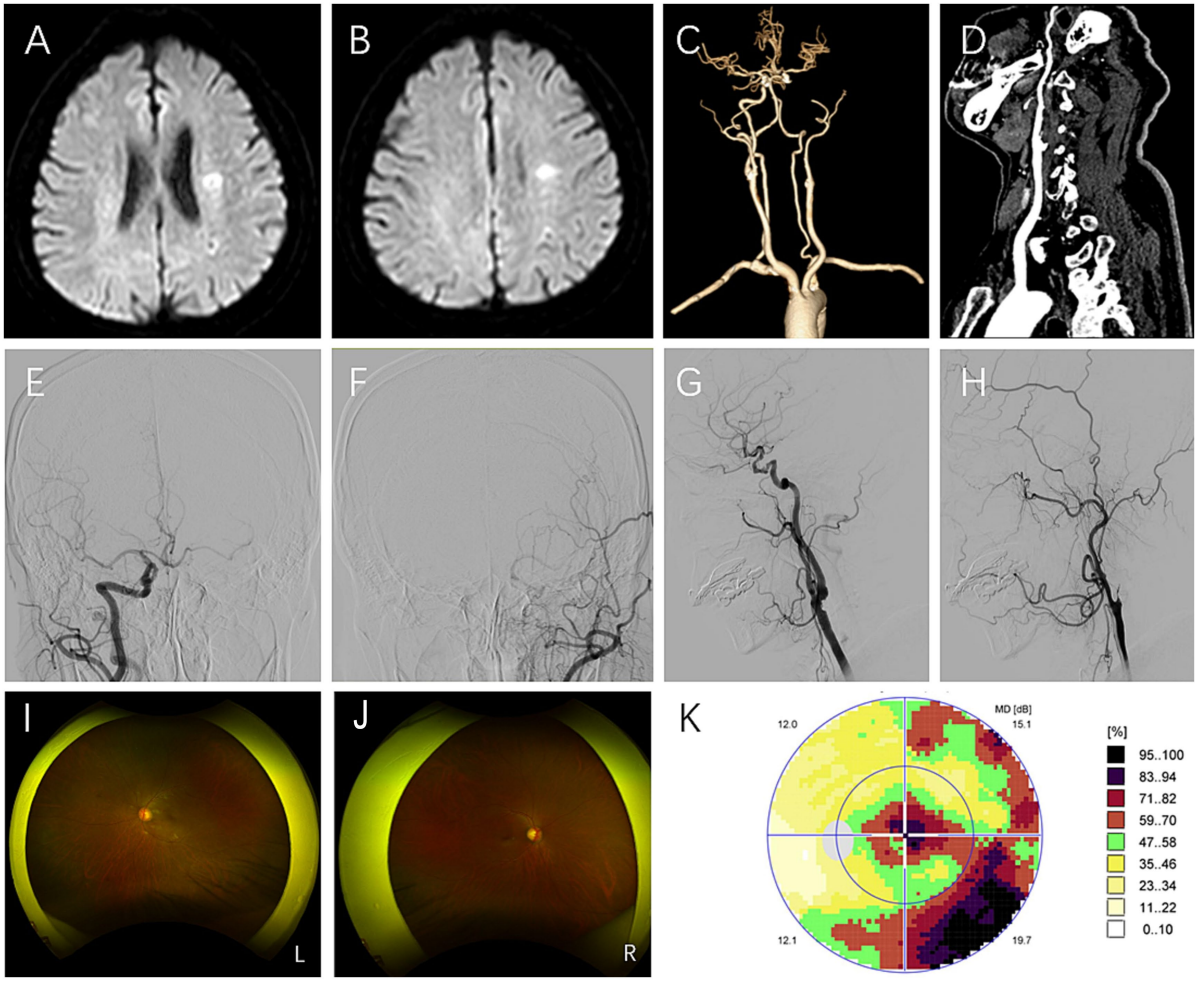
Imaging findings of Case 2. **(A,B)** DWI showing new infarcts in the left corona radiata and parietal lobe. **(C,D)** CTA showing occlusion of the left ICA origin. **(E–H)** DSA showing patent ICA stump with turbulent flow; distal vessel occluded. **(I–K)** Ophthalmic fluorescein fundus angiography showing a large avascular area in the left retina.

A 62-year-old man with diabetes mellitus was admitted with recurrent dizziness. MRI revealed an infarction in the left parietal lobe. Carotid ultrasonography showed no flow in the left ICA, and Computed tomography angiography (CTA) confirmed complete ICA occlusion. Dual antiplatelet therapy was initiated, but the patient did not take medication regularly after discharge. Two months later, dizziness recurred with amaurosis fugax in the left eye. Imaging showed a new infarction in the left corona radiata. Catheter angiography revealed a patent ICA stump with turbulent flow; the distal vessel was occluded. Retrograde ophthalmic artery collateralization and the characteristic stagnant flow pattern at the stump confirmed the chronic nature of the occlusion. Ophthalmic fluorescein fundus angiography showed a large avascular area in the left retina (Figures 2I–K). CSS was diagnosed. The family chose conservative treatment. At 3-month follow-up, no recurrent stroke occurred (Figure 2).

#### Case 3

**Figure 3:**
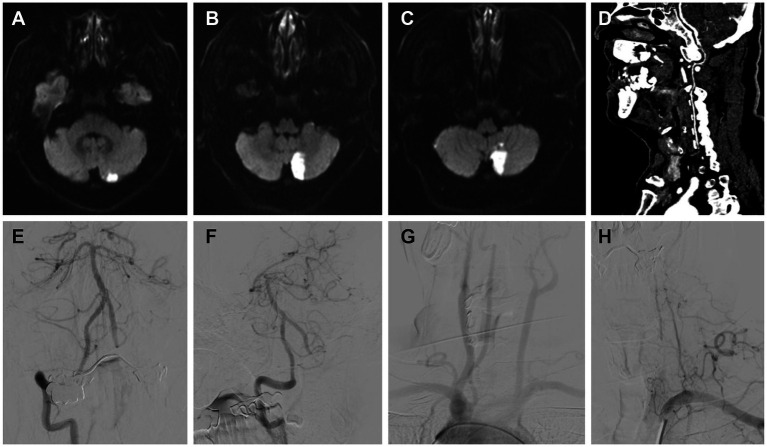
Imaging findings of Case 3. **(A–C)** DWI showing a new infarct in the left cerebellar hemisphere. **(D)** CTA showing occlusion of the left V1 segment. **(E–H)** DSA showing left V1 occlusion, with collateral flow from the ascending cervical artery and thyrocervical trunk and local turbulence.

A 62-year-old man with hypertension and coronary heart disease presented with sudden dizziness and gait deviation. MRI revealed an acute ischemic stroke in the left cerebellar hemisphere. CTA confirmed occlusion of the V1 segment of the left vertebral artery. Dual antiplatelet therapy (aspirin 150 mg and clopidogrel 75 mg once daily) was started. Catheter angiography showed occlusion of the left V1 segment, with collateral flow from the ascending cervical artery and thyrocervical trunk and local turbulence. This robust collateral network reconstituting the distal vertebral artery, together with the absence of fresh thrombus at the occlusion site, was indicative of chronic V1 segment occlusion. VASS was diagnosed. The regimen was changed to edoxaban 60 mg once daily. After gingival bleeding, the dose was reduced to 30 mg once daily, and bleeding resolved. At 1-year follow-up, no recurrent stroke occurred (Figure 3).

### Selection of studies for data extraction

**Figure 4:**
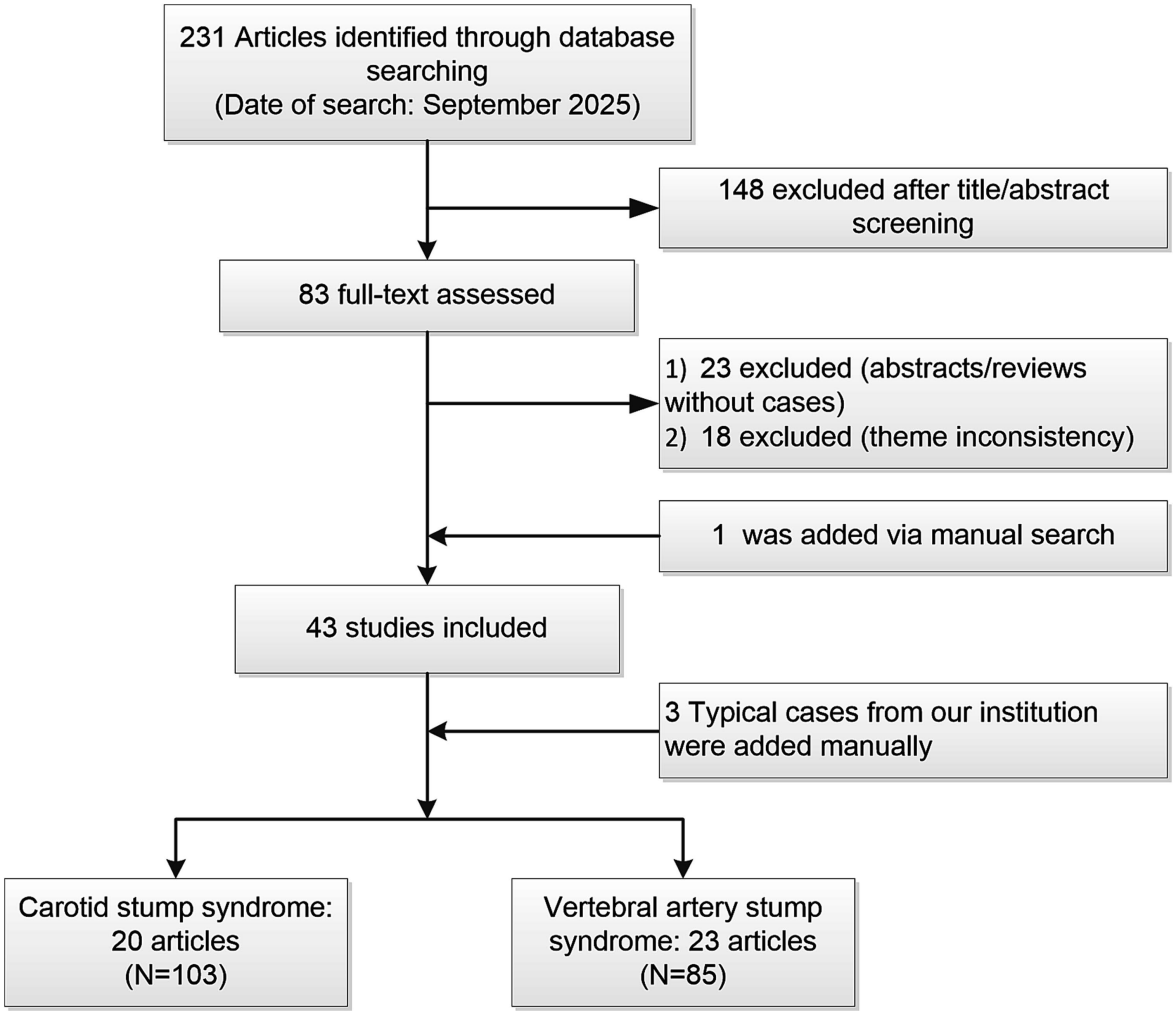
Study selection process of the review. Two hundred thirty-one records identified → 148 excluded after title/abstract screening → 83 full-text assessed → 23 excluded (abstracts/reviews without cases) → 60 assessed for eligibility → 18 excluded (theme inconsistency) → 42 from database search + 1 from manual search → 43 studies included.

A total of 231 studies were identified through the database searches. After title/abstract screening, 148 studies were excluded. Of the remaining 83 full-text articles, 23 abstracts or reviews without case reports were excluded. From the remaining 60, 18 were excluded because of inconsistency with the review theme. The research team checked reference lists and added one article via manual search. Finally, 43 studies were included (20 CSS-related [1, 3, 6–10, 15–17, 19–28] and 23 VASS-related [2, 4, 5, 11–13, 18, 29–44]), from which data of 188 patients were extracted (Figure 4).

### Baseline characteristics

All patients were diagnosed with stroke or transient ischemic attack caused by CASS using angiography. Among them, 76.1% (143/188) were male, median age at onset was 64.0 (56.0–69.0) years, and 53.4% (78/146) had recurrent neurological symptoms. Regarding treatment, 30.9% received medical treatment alone, 30.9% underwent carotid endarterectomy, 4.8% endovascular embolization, 29.8% endovascular recanalization, and 3.7% external carotid stenting. Median follow-up was 0.5 (0.3–3.4) years, with 8.0% (15/188) of experiencing recurrence (Table 1 the “Number of cases” column indicates available data per variable).

**Table 1:** Baseline characteristics of 188 patients with CASS confirmed using angiography.

Characteristics	Total (*N* = 188)	CSS (*N* = 103)	VASS (*N* = 85)	*p*-value	Number of cases
Baseline information					
Male, *n* (%)	143 (76.1%)	70 (68.0%)	73 (85.9%)	0.004	188
Age, median (IQR)	64.0 (56.0, 69.0)	62.0 (55.0, 67.0)	66.0 (57.0, 72.0)	0.005	188
Location of vascular occlusion, *n* (%)					
Left side	93 (60.4%)	41 (59.4%)	52 (61.2%)	0.825	154
Neurological symptoms, *n* (%)					
Recurrent episodes	78 (53.4%)	55 (53.4%)	23 (53.5%)	0.992	146
Amaurosis fugax	29 (25.2%)	29 (37.7%)	0 (0.0%)	<0.001	115
Vertigo	27 (23.5%)	3 (3.9%)	24 (63.2%)	<0.001	115
Hemianopsia	17 (14.8%)	2 (2.6%)	15 (39.5%)	<0.001	115
Ataxia	19 (16.5%)	2 (2.6%)	17 (44.7%)	<0.001	115
Conscious disturbance	23 (18.3%)	3 (3.9%)	20 (40.8%)	<0.001	126
Comorbidities, *n* (%)					
Hypertension	102 (75.6%)	45 (67.2%)	57 (83.8%)	0.024	135
Diabetes mellitus	41 (30.4%)	23 (34.3%)	18 (26.5%)	0.321	135
Treatment modality, *n* (%)					
Carotid endarterectomy	58 (30.9%)	58 (56.3%)	0 (0.0%)	<0.001	188
Pure medical treatment	58 (30.9%)	35 (34.0%)	23 (27.1%)	<0.001	188
Endovascular embolization	9 (4.8%)	2 (1.9%)	7 (8.2%)	<0.001	188
External carotid stenting	7 (3.7%)	7 (6.8%)	0 (0.0%)	<0.001	188
Endovascular recanalization	56 (29.8%)	1 (1.0%)	55 (64.7%)	<0.001	188
Outcomes					
Follow-up, median (IQR)	0.5 (0.3, 3.4)	2.0 (0.5, 4.0)	0.5 (0.3, 0.5)	<0.001	188
Recurrence, *n* (%)	15 (8.0%)	6 (5.8%)	9 (10.6%)	0.230	188

CASS, cerebral artery stump syndrome; CSS, carotid stump syndrome; IQR, interquartile range; VASS, vertebral artery stump syndrome; The ‘Number of cases’ column indicates the number of patients for whom data were available for each specific variable; totals vary due to incomplete reporting in the original studies.

Of the 188 patients, 103 had CSS and 85 VASS. Compared with the VASS group, the CSS group had a lower proportion of males (68.0% vs. 85.9%, *p* = 0.004) and a younger median age (62.0 vs. 66.0 years, *p* = 0.005). Amaurosis fugax occurred in 25.2% of CSS patients; vertigo (3.9% vs. 63.2%, *p* < 0.001), hemianopsia (2.6% vs. 39.5%, p < 0.001), ataxia (2.6% vs. 44.7%, *p* < 0.001), and consciousness disturbance (3.9% vs. 40.8%, *p* < 0.001) were less frequent in CSS than in VASS. Hypertension was less prevalent in CSS (67.2% vs. 83.8%, *p* = 0.024). CSS Patients were more likely to receive medical treatment alone (34.0% vs. 27.1%, *p* < 0.001), carotid endarterectomy (56.3% vs. 0.0%, *p* < 0.001), and external carotid stenting (6.8% vs. 0.0%, *p* < 0.001), and less likely to undergo endovascular embolization (1.9% vs. 8.2%, *p* < 0.001) or endovascular recanalization (1.0% vs. 64.7%, *p* < 0.001). Follow-up was longer for CSS (2.0 vs. 0.5 years, *p* < 0.001). Recurrence rates did not differ significantly (5.8% vs. 10.6%, *p* = 0.230) (Table 1).

### Recurrence outcome characteristics

Comparison between the 15 patients with recurrence and 173 without recurrence showed that therapeutic modality was the main factor associated with recurrence. The recurrence group had a higher proportion of patients who receiving medical treatment alone (73.3% vs. 27.2%, *p* = 0.015), and lower proportions receiving carotid endarterectomy (6.7% vs. 32.9%, *p* = 0.015), endovascular embolization (0.0% vs. 5.2%, *p* = 0.015), external carotid artery stenting (0.0% vs. 4.0%, *p* = 0.015), and endovascular recanalization (20.0% vs. 30.6%, *p* = 0.015). Follow-up time was similar between groups (0.5 vs. 0.5 years, *p* = 0.330) (Table 2).

**Table 2:** Recurrence outcome characteristics of 188 patients with CASS confirmed using angiography.

Characteristics	No recurrence (*N* = 173)	Recurrence (*N* = 15)	*P*-value
Baseline information			
Male, *n* (%)	129 (74.6%)	14 (93.3%)	0.124
Age, median (IQR)	64.0 (56.0, 69.0)	60.0 (55.0, 66.0)	0.233
Location of vascular occlusion, *n* (%)			
Left side	86 (61.4%)	7 (50.0%)	0.404
Neurological symptoms, *n* (%)			
Recurrent episodes	69 (51.5%)	9 (75.0%)	0.118
Amaurosis fugax	26 (25.2%)	3 (25.0%)	>0.999
Vertigo	22 (21.4%)	5 (41.7%)	0.149
Hemianopsia	13 (12.6%)	4 (33.3%)	0.077
Ataxia	13 (12.6%)	6 (50.0%)	0.005
Conscious disturbance	20 (17.9%)	3 (21.4%)	0.719
Comorbidities, *n* (%)			
Hypertension	91 (74.0%)	11 (91.7%)	0.293
Diabetes mellitus	38 (30.9%)	3 (25.0%)	>0.999
Treatment modality, *n* (%)			
Carotid endarterectomy	57 (32.9%)	1 (6.7%)	0.015
Pure medical treatment	47 (27.2%)	11 (73.3%)	0.015
Endovascular embolization	9 (5.2%)	0 (0.0%)	0.015
External carotid stenting	7 (4.0%)	0 (0.0%)	0.015
Endovascular recanalization	53 (30.6%)	3 (20.0%)	0.015
Outcomes			
Follow-up, median (IQR)	0.5 (0.3, 4.0)	0.5 (0.3, 1.0)	0.330

CASS, cerebral artery stump syndrome; IQR, interquartile range.

Among the 188 patients, the recurrence rate with medical treatment alone was 19.0%, which was significantly higher than that of invasive treatments (carotid endarterectomy 1.7%, endovascular embolization 0.0%, external carotid artery stenting 0.0%, and endovascular recanalization 5.4%; p = 0.015). Among the 76 patients (40.4%) with complete medication data, the recurrence rate with initial antiplatelet therapy (93.0%) was significantly higher than that with initial anticoagulant therapy (42.1%, *p* < 0.001) (Supplementary Figure 1). Of the 19 patients in the initial anticoagulant therapy group, 8 (42.1%) recurred. Subsequently, one patient underwent endovascular embolization, one underwent endovascular recanalization, and four underwent carotid endarterectomy (no further recurrences), while two who continued the original anticoagulant therapy still recurred. Among the 57 patients in the initial antiplatelet therapy group, 53 (93.0%) recurred. Subsequently, three underwent endovascular recanalization, seven external carotid stenting, and eight endovascular embolization (no recurrences), 24 received carotid endarterectomy (23 no recurrence, 1 recurrence), and 11 continued antiplatelet (4 no recurrence, 7 recurrence).

### Procedural complications of invasive treatments

Procedural complications extracted from the original studies are summarized as follows. Carotid endarterectomy was associated with 2 cases of new-onset myocardial infarction, 1 case new cerebral infarction, 1 case wound hematoma, and 1 case cranial nerve palsy. External carotid stenting resulted in 3 cases of transient neurological deficits. Endovascular recanalization was related to 3 cases of distal embolization, 1 case punctate hemorrhagic transformation, 1 case asymptomatic vascular dissection, and 1 case dead caused by reperfusion edema. No complications were reported for endovascular embolization.

### Exploratory era-stratified analysis

A post-hoc analysis stratifying patients by era of publication (1978–2000, 2001–2010, 2011–2025) showed no statistically significant difference in recurrence rates across eras (Supplementary Table S1), although the analysis was underpowered.

## Discussion

This study is the first to summarize the baseline characteristics, therapeutic strategies, and prognostic differences of patients with CASS, clarify the impact of different treatment regimens on disease recurrence, and provide an evidence-based foundation for the individualized clinical diagnosis and treatment of CASS. Our findings revealed that therapeutic modality is the key factor associated with recurrence in patients with CASS. The recurrence rate for medical treatment alone was significantly higher than that for invasive treatments (carotid endarterectomy, endovascular embolization, external carotid stenting, and endovascular recanalization), with a recurrence rate as high as 19.0% for medical treatment alone. Further subgroup analysis of medical treatment alone showed a disease recurrence rate with initial anticoagulant therapy (42.1%) substantially lower than that with initial antiplatelet therapy (93.0%). Only one case of recurrent stroke occurred in a patient who underwent invasive treatment after failure of medical treatment.

The differences in therapeutic efficacy observed in this study stem from the core characteristics of its pathophysiological mechanism and anatomical differences between subtypes. First, the core pathogenesis of CASS involves thrombus formation caused by turbulent blood flow at the occluded vascular stump or collateral circulation area and artery-to-artery embolism induced by thrombus detachment, which is the fundamental cause of recurrent ischemic events (10, 11). Medical treatment alone can only inhibit thrombus formation through antithrombotic effects but cannot eliminate turbulent flow and block the embolus source at its origin, resulting in a high risk of recurrence. In contrast, invasive treatments directly intervene at the lesion site to eliminate the source of emboli. For example, carotid endarterectomy occludes the CSS stump, endovascular recanalization reconstructs the VASS blood flow to eliminate turbulence, endovascular embolization blocks the embolus pathway of collateral circulation, and external carotid stenting isolates the CSS stump; all of these interventions achieve effective occlusion of the embolus source, thus being associated with a significant lower recurrence rate (9, 11–13). Second, thrombus formation in patients with CASS is caused by blood stasis induced by turbulent blood flow; the resulting thrombus is predominantly a red thrombus rather than the white thrombus caused by atherosclerotic plaque rupture (14). Anticoagulant act on multiple links of the coagulation cascade to effectively inhibit fibrin formation and specifically block red thrombus generation. Conversely, antiplatelet drugs only inhibit platelet aggregation, with limited effect on red thrombi, thus leading to a significantly higher recurrence rate with initial antiplatelet therapy. Our observation that anticoagulation was associated with lower recurrence than antiplatelet therapy is consistent with the hypothesis that stasis-related thrombi in CASS may be predominantly red (fibrin-rich) thrombi. However, this interpretation remains speculative based on our retrospective data and should be considered hypothesis-generating. Direct histopathological evidence from CASS patients is needed to confirm the thrombus composition.

The CSS was first reported by Barnett et al. (1), with the core feature of recurrent anterior circulation ischaemia caused by the stump after ICA occlusion and amaurosis fugax as a typical symptom. In contrast, VASS was first proposed by Nguyen et al. in 2008, accounting for 1.4% of acute posterior circulation ischemic strokes. All patients had cerebellar infarction with vertigo and ataxia as the main manifestations (2). In this study, we verified these characteristics using large-sample data and further quantified the differences in clinical symptoms between the CSS and VASS. Regarding therapeutic efficacy, existing studies suggest that carotid endarterectomy for CSS effectively occludes the stump and blocks the embolus source, with only one case of recurrent stroke among 25 patients with CSS who underwent surgery; in addition, the technical success rate of endovascular recanalization for VASS reaches 86–96.7% with a low incidence of perioperative complications (7, 13). Our results are consistent with these findings, showing a recurrence rate of 6.7% for carotid endarterectomy for CSS, and confirming that endovascular recanalization is widely used and has definite efficacy for VASS. Existing studies have proposed that anticoagulant therapy is superior to antiplatelet therapy for CASS but have not quantified the difference in recurrence (4). Based on data from 76 patients who received complete medication regimens, this study clarified for the first time that the recurrence rate with initial anticoagulant therapy is considerably lower than that with antiplatelet therapy, providing a quantitative basis for clinical drug selection. Additionally, there are few reports on the application of external carotid stenting and endovascular embolization in CASS in existing studies. The present study found no recurrence with these two invasive treatment modalities, supplementing the efficacy data for CASS treatment.

### Temporal heterogeneity and publication bias

The pooled studies span nearly five decades (1978–2025). Diagnostic criteria, imaging technology (from conventional angiography to CTA/MRA), and treatment standards (including newer antiplatelets and DOACs) have evolved substantially. Moreover, case reports inherently favor unusual or successful outcomes, potentially inflating the apparent efficacy of interventions. Our exploratory era-stratified analysis did not show a statistically significant difference in recurrence rates across eras, but this analysis is underpowered. Publication bias (over-representation of successful interventions) may still overestimate the benefits of invasive treatments and anticoagulation. These issues are important limitations, as discussed below.

### Limitations

This study has several limitations. First, treatment allocation was not random, and confounding by indication is highly likely. Patients receiving medical treatment alone may have differed systematically from those receiving invasive treatment (e.g., surgical candidacy, comorbidity burden, institutional resources, era of treatment). Residual confounding cannot be excluded. Second, publication bias is present: included studies are mostly case reports and small case series that tend to report favorable outcomes. Third, clinical data were incomplete: only 40.4% of patients had complete medication records, and details such as stump morphology, occlusion length, medication compliance, and postoperative antithrombotic regimens were not systematically reported. Fourth, the follow-up period was short (median 0.5 years), limiting assessment of long-term prognosis. Fifth, procedural complications are likely underreported in the original studies. Sixth, the small number of outcome events (n = 15) precluded multivariable or propensity-matched analyses.

## Conclusion

The CASS is a rare cerebrovascular disease with a core mechanism of thromboembolism caused by abnormal blood flow in the stump or collateral circulation. CSS and VASS exhibit significant subtype-specific differences in clinical features and therapeutic strategies. The core treatment is embolus source occlusion. Invasive treatment was associated with a lower recurrence rate than medical treatment alone, and anticoagulant therapy is the first choice for medical treatment. In the future, large-scale, prospective, multicenter studies are needed to validate these findings and explore individualized diagnosis and treatment strategies to provide a stronger evidence-based foundation for the standardized clinical diagnosis and treatment of CASS.

## Data Availability

The original contributions presented in the study are included in the article/Supplementary material, further inquiries can be directed to the corresponding author.
