# Supplementary material for: Cerebral artery stump syndrome: a comprehensive pooled analysis of 188 angiographically confirmed cases
Source: Front Neurol. 2026 Jul 7;17:1840765. doi: 10.3389/fneur.2026.1840765 (PMC13384845; doi:10.3389/fneur.2026.1840765)

| **Supplementary Table S1.** Exploratory era stratified analysis. | | | | | |
| --- | --- | --- | --- | --- | --- |
| Characteristics | 1978-2000  (N=11) | 2001-2010  (N=33) | 2011-2025  （N=144） | P value | Number of cases |
| Baseline information | | | | | |
| Male, n (%) | 7 (63.6%) | 26 (78.8%) | 110 (76.4%) | 0.602 | 188 |
| Age, median (IQR) | 53.0 (51.0, 64.0) | 61.0 (55.0, 66.0) | 65.0 (57.5, 70.0) | 0.026 | 188 |
| Location of vascular occlusion, n (%) | | | | | |
| Left side | 1 (50.0%) | 5 (62.5%) | 87 (60.4%) | >0.999 | 154 |
| Neurological symptoms, n (%) | | | | | |
| Recurrent episodes | 8 (72.7%) | 20 (60.6%) | 50 (49.0%) | 0.209 | 146 |
| Amaurosis fugax | 5 (45.5%) | 11 (33.3%) | 13 (18.3%) | 0.051 | 115 |
| Vertigo | 0 (0.0%) | 5 (15.2%) | 22 (31.0%) | 0.025 | 115 |
| Hemianopsia | 0 (0.0%) | 5 (15.2%) | 12 (16.9%) | 0.446 | 115 |
| Ataxia | 0 (0.0%) | 4 (12.1%) | 15 (21.1%) | 0.214 | 115 |
| Conscious disturbance | 0 (0.0%) | 3 (9.1%) | 20 (24.4%) | 0.045 | 126 |
| Comorbidities, n (%) | | | | | |
| Hypertension | 1 (50.0%) | 2 (66.7%) | 99 (76.2%) | 0.355 | 135 |
| Diabetes mellitus | 0 (0.0%) | 2 (66.7%) | 39 (30.0%) | 0.291 | 135 |
| Treatment modality, n (%) | | | | | |
| Carotid endarterectomy | 8 (72.7%) | 25 (75.8%) | 25 (17.4%) | - | 188 |
| Pure medical treatment | 3 (27.3%) | 3 (9.1%) | 52 (36.1%) | - | 188 |
| Endovascular embolisation | 0 (0.0%) | 2 (6.1%) | 7 (4.9%) | - | 188 |
| External carotid stenting | 0 (0.0%) | 3 (9.1%) | 4 (2.8%) | - | 188 |
| Endovascular recanalisation | 0 (0.0%) | 0 (0.0%) | 56 (38.9%) | - | 188 |
| Outcomes | | | | | |
| Follow-up, median (IQR) | 0.8 (0.2, 1.5) | 0.5 (0.3, 1.0) | 0.5 (0.5, 4.0) | 0.161 | 188 |
| Recurrence, n (%) | 1 (9.1%) | 2 (6.1%) | 12 (8.3%) | >0.999 | 188 |

**Supplementary Figure1.**


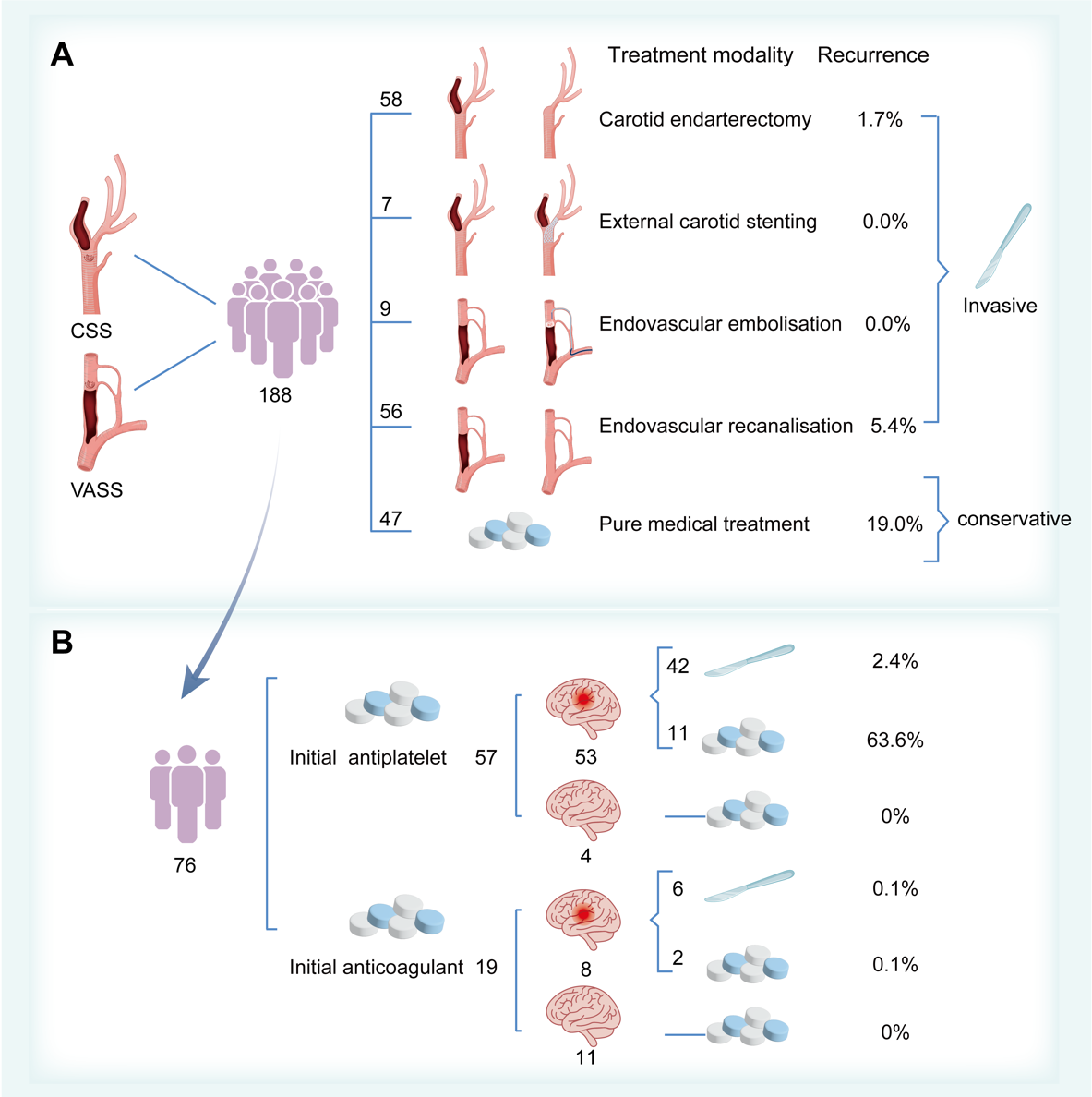

Supplement: Supplementary file 1 [file Table_1.docx]
